# Adenosine A2A receptor signaling promotes FoxO associated autophagy in chondrocytes

**DOI:** 10.1038/s41598-020-80244-x

**Published:** 2021-01-13

**Authors:** Benjamin Friedman, Carmen Corciulo, Cristina M. Castro, Bruce N. Cronstein

**Affiliations:** 1 Department of Medicine, Division of Rheumatology, NYU School of Medicine, 550 First Avenue, New York, NY 10016 USA; 2 Department of Medicine, Division of Translational Medicine, NYU School of Medicine, 550 First Avenue, New York, NY 10016 USA

**Keywords:** Biochemistry, Cell biology, Immunology, Molecular biology, Physiology, Medical research, Molecular medicine, Rheumatology

## Abstract

Autophagy, a homeostatic pathway upregulated during cellular stress, is decreased in osteoarthritic chondrocytes and this reduction in autophagy is thought to contribute to the development and progression of osteoarthritis (OA). The adenosine A2A receptor (A2AR) is a potent anti-inflammatory receptor and deficiency of this receptor leads to the development of OA in mice. Moreover, treatment using liposomally conjugated adenosine or a specific A2AR agonist improved joint scores significantly in both rats with post-traumatic OA (PTOA) and mice subjected to a high fat diet obesity induced OA. Importantly, A2AR ligation is beneficial for mitochondrial health and metabolism in vitro in primary and the TC28a2 human cell line. An additional set of metabolic, stress-responsive, and homeostatic mediators include the Forkhead box O transcription factors (FoxOs). Data has shown that mouse FoxO knockouts develop early OA with reduced cartilage autophagy, indicating that FoxO-induced homeostasis is important for articular cartilage. Given the apparent similarities between A2AR and FoxO signaling, we tested the hypothesis that A2AR stimulation improves cartilage function through activation of the FoxO proteins leading to increased autophagy in chondrocytes. We analyzed the signaling pathway in the human TC28a2 cell line and corroborated these findings in vivo in a metabolically relevant obesity-induced OA mouse model. We found that A2AR stimulation increases activation and nuclear localization of FoxO1 and FoxO3, promotes an increase in autophagic flux, improves metabolic function in chondrocytes, and reduces markers of apoptosis in vitro and reduced apoptosis by TUNEL assay in vivo. A2AR ligation additionally enhances in vivo activation of FoxO1 and FoxO3 with evidence of enhanced autophagic flux upon injection of the liposome-associated A2AR agonist in a mouse obesity-induced OA model. These findings offer further evidence that A2AR may be an excellent target for promoting chondrocyte and cartilage homeostasis.

## Introduction

Osteoarthritis (OA) is a common medical condition characterized by degeneration of articular cartilage and subchondral bone that affects approximately 25% of adults over age 65^1^. Currently there are no successful medical approaches for treating or preventing OA and it is thus an important area for medical research in order to reduce patient morbidity and economic costs. The factors most commonly cited as contributing to development of OA include advanced age, obesity, prior joint trauma, genetics, and anatomic factors^2,3^. Aging^3,4^ and obesity^5^ represent the two most important risk factors for OA development leading to cartilage destruction in part via shear forces in load-bearing joints as well as deleterious alterations in cellular metabolism of chondrocytes or various inflammatory cells^6^.

Various changes in articular cartilage observed with OA include reduced chondrocyte cellularity and degradation of cartilage extracellular matrix (ECM) proteins including type II collagen and aggrecan secondary to increases in matrix degrading enzymes^4^. In OA chondrocytes there are alterations in various homeostatic mechanisms including: reduced autophagy, increased reactive oxygen species (ROS) production, and reduced mitochondrial function. Autophagy functions as a protective energy-conserving mechanism that recycles dysfunctional organelles or macromolecules into their constituent molecules in times of cell stress and starvation^7^. During autophagy, dysfunctional protein cargo is enveloped by newly formed double-membraned vesicles termed autophagosomes that fuse with lysosomes resulting in lysosomal breakdown of autophagosome cargo^8^. The protein p62/SQSTM1 plays an important role in autophagy by simultaneously binding to both ubiquitinated autophagosomal cargo constituents and autophagosome membrane-associated mammalian *Atg8* homologs including *LC3*, *Gabarap*, *Gabarapl1*, and *Gabarapl2*^9–11^. Importantly for experimental analysis, p62 is directly substrate bound and therefore is degraded on lysosomal fusion as autophagy proceeds. Unlike p62, Atg8 homologs LC3 and Gabarapl1 are generally recycled as they are delipidated from the membrane after a cycle of autophagy^12,13^. Specifically, Gabarapl1 interacts with both key upstream canonical autophagosome signaling complexes, is involved in autophagosome-lysosome fusion, and is included in the mitochondrial-specific autophagy (mitophagy) pathway defined by the Kyoto Encyclopedia of Genes and Genomes (KEGG, pathway hsa04137)^11,14,15^. The more typically employed autophagy marker LC3B is not listed directly in this mitophagy KEGG pathway^14^. Moreover, Gabarap, Gabarapl1, and Gabarapl2 are required for both starvation-induced autophagy and mitophagy whereas LC3 proteins are not^16,17^.

There are numerous upstream mediators of the autophagy pathway such as the signaling kinases mammalian target of rapamycin (mTOR) and AMP-Activated Protein Kinase (AMPK) as well as transcription factors including pro-autophagy, organism longevity-associated Forkhead Box (FoxO) transcription factors. Recent work by *Matsuzaki *et al*.* demonstrated that FoxO1/3/4 triple knockout mice exhibited histologic OA cartilage changes, reduction in cartilage PRG4 (lubricin) protein levels, as well as decreases in numerous autophagy and stress responsive genes. These changes were most discernible in FoxO1 knockout mice followed by FoxO3 knockouts^18^. Specifically, the authors found that FoxO1 knockout mice developed severe OA cartilage lesions by 6 months and FoxO3 cartilage defects occurred in older mice by 18 months. Additionally, there was increased autophagy gene expression in mouse and human OA primary chondrocytes with FoxO1 overexpression^18^.

Regulation of FoxO transcription factors is a complex process involving numerous activating and inhibiting modifications including protein phosphorylation and acetylation. Akt/PKB phosphorylation of FoxO1 and FoxO3 leads to inactivation and nuclear exclusion^19^. Non-Akt phosphorylated FoxO1/3 can be transported into the nucleus to activate transcription, whereas Akt-phosphorylated FoxOs contained in the nucleus of quiescent cells can be exported by the carrier protein 14-3-3 and degraded by the ubiquitin-proteosomal pathway^20–22^.

Adenosine is a purine nucleoside derived predominantly from ATP hydrolysis that is produced in response to inflammatory stimuli, hypoxia, or metabolic stress^23^. It functions extracellularly by differentially binding four G-protein coupled receptors (GPCRs): A1R, A2AR, A2BR, and A3R. Signaling through A2AR, a G_s_ associated GPCR, has been shown to be essential to chondrocyte homeostasis; mice lacking A2AR spontaneously develop OA^24^. Additionally, intra-articular injection of liposomal preparations of adenosine prevents the development of post-traumatic OA (PTOA) after ACL rupture in rats^24^. The effect of liposomal adenosine on development of OA is mediated through ligation of the A2AR since an A2AR antagonist, but not A2BR or A3R antagonists, completely reverses the effect of adenosine on OA in this model. Importantly, the beneficial, cartilage regenerative changes observed with A2AR ligation in PTOA rats was paralleled in knee joints from an obesity (high fat diet induced) OA mouse model treated with injections of liposomal adenosine or an A2AR-specific agonist^25^. Furthermore, results from our lab have highlighted A2AR signaling as essential for mitochondrial structure and function as well as reducing IL-1ß induced chondrocyte oxidative stress in human primary cells with very similar effects observed in cells from the TC28α2 human chondrocyte cell line.

Recent differential expression studies from our lab comparing chondrocytes from A2AR knockout and WT mice (unpublished observation) and cartilage from post-traumatic OA (PTOA) treated with or without joint injections of liposomal A2AR agonist suggest that A2AR ligation might prevent OA progression by stimulating reparative mechanisms, including autophagy, that collectively promote chondrocyte homeostasis^25^. We therefore tested the hypothesis that A2AR stimulation increases autophagy through FoxO signaling in chondrocytes and thereby maintains chondrocyte homeostasis. We report here that, indeed, A2AR ligation stimulates activation and nuclear localization of FoxO1 and FoxO3 resulting in increased expression of the autophagic proteins p62/SQSTM1, Gabarapl1 and Beclin-1 and enhanced autophagic flux in vitro. Similar changes were observed in vivo in cartilage from a murine model of obesity-induced OA treated with an A2AR agonist.

## Results

### A2AR stimulation leads to nuclear translocation and increased total cellular and nuclear protein levels of autophagy transcriptional mediators FoxO1 and FoxO3 in vitro

**Figure 1:**
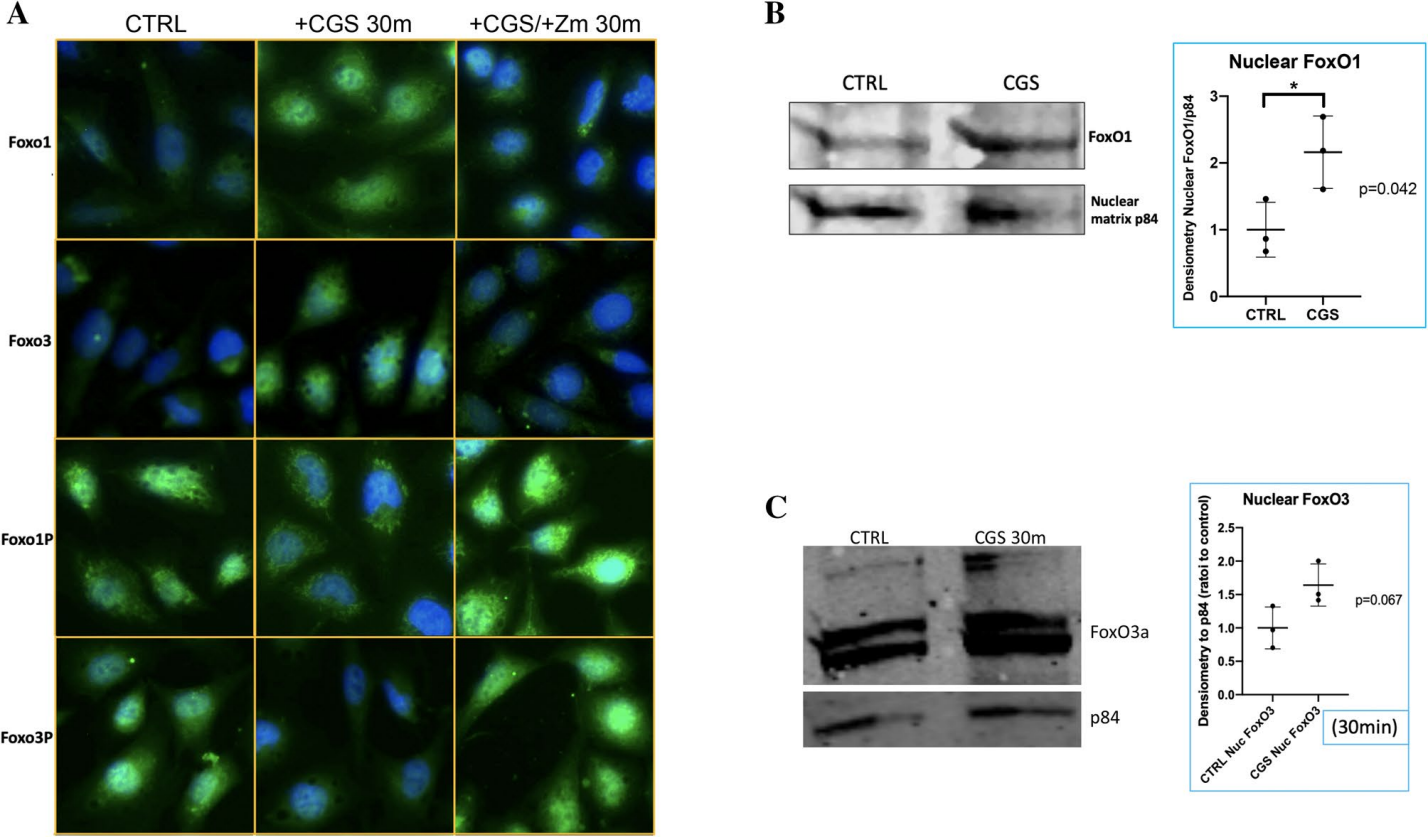
A2AR binding promotes FoxO1 and FoxO3 nuclear translocation and significant increase in nuclear FoxO1 levels. (**A**) Different groups of TC28α2 cells labeled as CTRL (untreated chondrocytes), + CGS for 30 m (treated), and + CGS/ + Zm (pre-treated with the A2AR antagonist). FoxO1, FoxO3, FoxO1-P (Ser256), FoxO3-P (Ser253) are displayed in green by IF for these various cell conditions at 40 × hpf. These cell images were representative of 3–4 individual experiments for each antibody and condition. DAPI is in blue, and nuclear overlap appears aqua-bright or green in the nuclear compartment. (**B**) Western blot of the nuclear fraction protein extract from TC28⍺2 human chondrocytes treated with or without 1 µM CGS for 30 m assessing FoxO1 protein levels with nuclear protein loading control nuclear matrix protein p84. Calculation of nuclear FoxO1/p84 densitometry (normalized to nuclear control) in individual western blotting experiments with CGS vs. control 2.2 ± 0.5 vs. 1.0 ± 0.4 (mean ± standard deviation), *p < 0.05 by unpaired t-test, n = 3 per group. (**C**) Western blot of the nuclear fraction protein extract from TC28⍺2 human chondrocytes treated with or without 1 µM CGS for 30 m assessing FoxO3 protein levels with nuclear protein loading control nuclear matrix protein p84.

We first determined pro-autophagy transcription factors FoxO1 and FoxO3 as well as their inactive phosphorylated forms phospho-FoxO1 (Ser 256) and phospho-FoxO3 (Ser 253) in TC28α2 chondrocytic cells. To assess the localization of active and inactive FoxOs, we performed IF in TC28α2 chondrocytes and observed that CGS21680 induced nuclear translocation of both FoxO1 and FoxO3 along with nuclear export of inactive phosphorylated FoxOs (labeled FoxO1-P and FoxO3-P) and these effects were reversed by pre-treatment with the A2AR antagonist ZM241385, 1 µM (Fig. 1A). Importantly, the majority of the A2AR-activated increase in nuclear FoxO1 and FoxO3 is most likely in its active form given the increase in FoxO1/3 with concomitant nuclear decrease in the inactive Akt-phosphorylated FoxO1 and FoxO3 forms. We also assessed FoxO1 and FoxO3 levels in TC28α2 chondrocytes treated with or without 1 µM CGS21680 by western blot and found significantly elevated nuclear levels of FoxO1 and near-signficant elevation in nuclear FoxO3 in A2AR agonist treated cells (Fig. 1B,C). Analysis of total cellular levels of total FoxO1 and FoxO3 at 0, 15, and 30 min showed an upward trend for each in TC28a2 cells (Fig. S2A,B). These results are consisten with an in vitro A2AR-mediated activation of FoxO1 and FoxO3 transcription factors in chondrocytes. A2AR ligation also induced a significant decrease in inactive phosphorylated FoxO1 in total cellular protein extracted from TC28a2 cells (Fig. S2C); total cellular phospho-FoxO3 was similar for the untreated and treated TC28a2 cells (Fig. S2D).

### A2AR stimulation promotes autophagic flux in chondrocytes in vitro in a FoxO-mediated fashion

**Figure 2:**
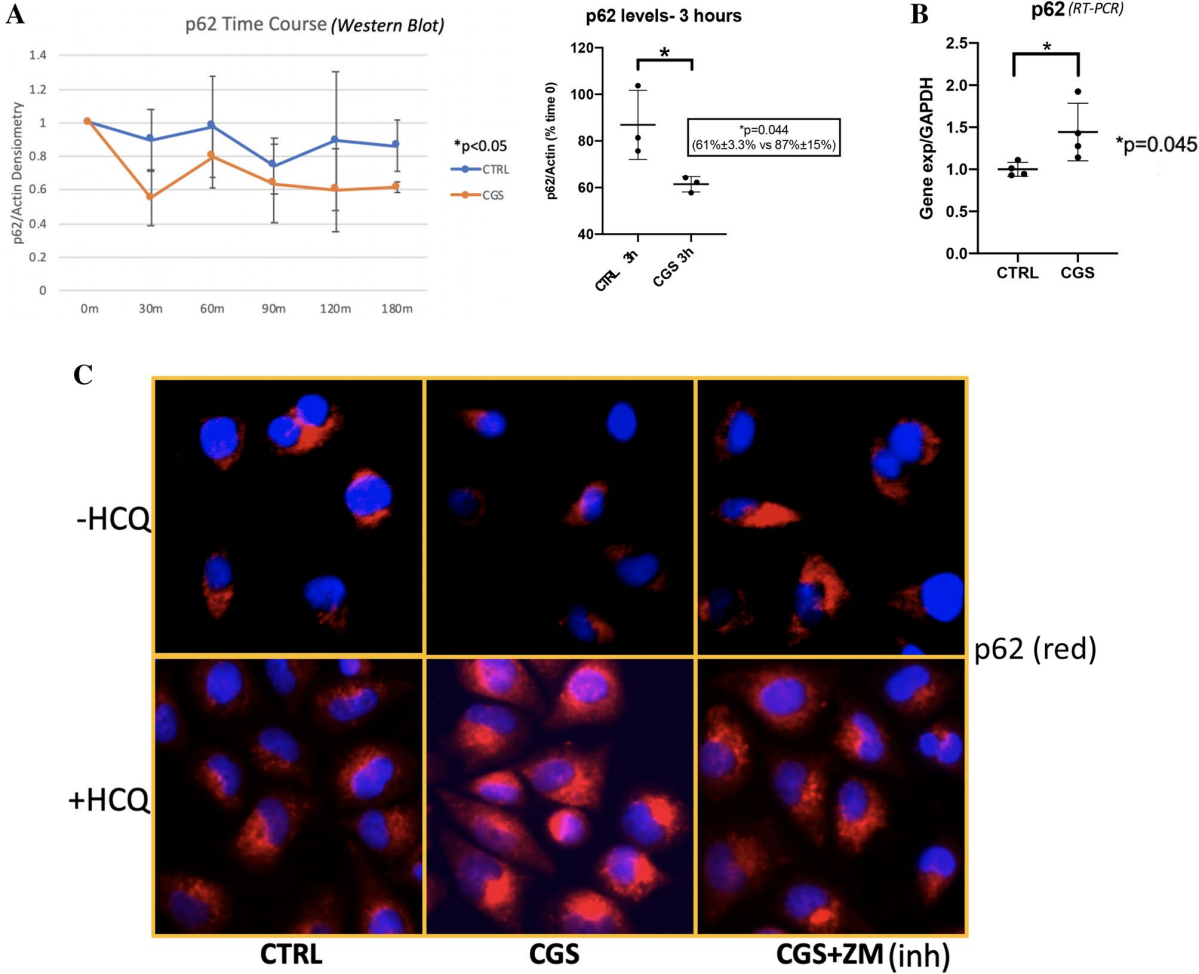
Autophagy protein p62 analysis shows A2AR agonism increases autophagic flux. Of note, p62 protein is directly bound to cargo in the autophagosome leading to its degradation by lysosomal proteases. Hence, its degradation can be used as a positive marker of autophagy. (**A**) Western blot time course for p62 levels from TC28⍺2 cells all in starvation media with FBS reduced from 10 to 1% and treated ± 1 µM CGS with line graph trend below (+ CGS, blue line, -CGS, orange line). Densitometry of p62 was compared to that of actin and normalized to time 0 are plotted for the time points with time course analysis by time series mixed model ANOVA (time series CGS vs. CTRL 0.74 ± 0.17 vs. 1.0 ± 0.24, * p < 0.05, n = 3 per time point per group). Individual comparison of 3-h CGS vs. CTRL sample sets showed statistically significant percent decrease in p62 by 3 h compared to time 0 in treated vs. untreated (61% ± 3.3% vs. 87% ± 15%, *p* = 0.044, n = 3). (**B**) p62 mRNA levels were measured in vitro after CGS treatment. CGS vs. CTRL was significantly increased (1.44 ± 0.34 vs. 1.0 ± 0.082, *p* = 0.045, n = 4). (**C**) TC28⍺2 human chondrocytes were stained for p62 (red) by IF at 1 h after CGS treatment. CTRL, untreated; CGS, treated A2AR agonist; CGS + ZM, A2AR antagonist pre-treatment with A2AR agonist treatment. Representative cells at 40 × HPF from 4 repeated experiments per condition.

Treatment of TC28α2 chondrocytes with 1 µM CGS21680 in serum-depleted media (FBS 1%) leads to a more pronounced decrease in p62 protein levels at all time points assessed over 3 h compared to starvation-induced autophagy alone as observed in the CGS21680 vs. control time course (Fig. 2A). Comparison of CGS and CTRL samples at 3 h showed a significant reduction in p62 with A2AR stimulation with levels of 61% of time 0 p62 levels in the CGS21680-treated vs. 87% in the control serum-starved cells (Fig. 2A). Similar A2AR-mediated p62 decrease also occurred in fully supplemented media. Notably, p62 gene expression increased significantly 1.44-fold in CGS-treated cells (Fig. 2B). While this is just a moderate change, this transcriptional increase may actually indicate underestimation of p62 protein degradation as an indicator of autophagic flux, thus further accentuating the difference observed in Fig. 2A.

The reduction in cellular p62 was also detectable by immunofluorescence (IF) after 1 h of treatment with 1 µM CGS21680, and the reduction was reversible by pre-treatment with the A2AR antagonist ZM241385 (Fig. 2C, top row). When fusion of the autophagosomes with the lysosomes was inhibited by overnight pre-treatment with hydroxychloroquine (HCQ) we observed accumulation of p62 in unfused autophagosomes, as previously reported^26,27^. In the presence of HCQ there was a clear increase in autophagosomal p62 levels in chondrocytes treated with 1 µM CGS21680 compared to untreated cells. Moreover, this increase in the presence of HCQ was mostly reversible by pre-treatment of cells with the A2AR antagonist ZM241385 (Fig. 2C, bottom row). These results indicate that A2AR activation stimulates p62-mediated autophagic flux.

**Figure 3:**
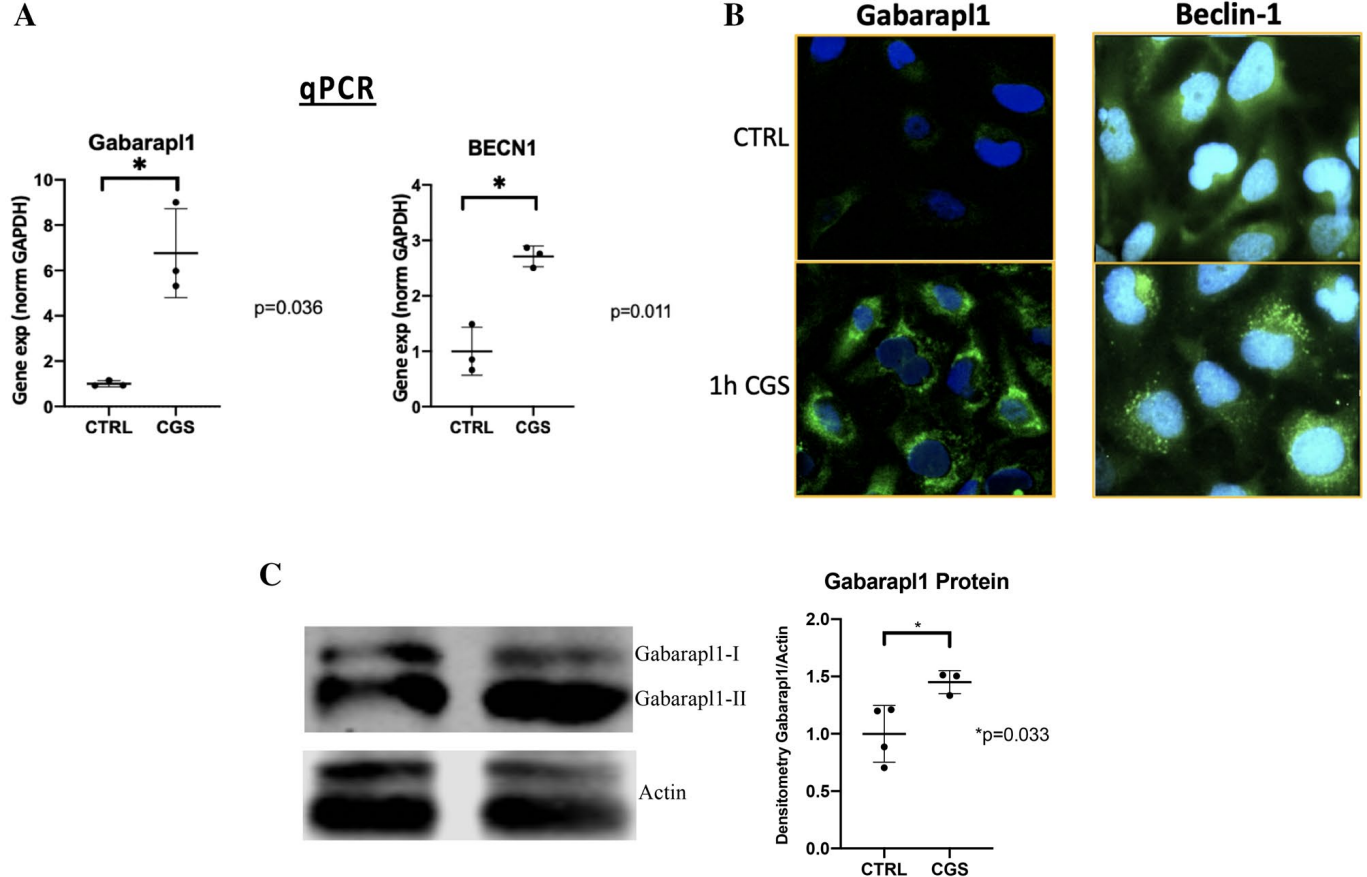
FoxO-activated autophagy mediators Gabarapl1, and Beclin-1 were activated in response to A2AR signaling. TC28⍺2 human chondrocytes were treated with 1 µM CGS (Gabarapl1-assessed cells were incubated with HCQ overnight to enhance autophagosome visualization). (**A**) RNA was isolated from cells and Gabarapl1 and Beclin-1 gene expression were assessed. (**B**) Autophagy proteins were visualized by IF in TC28⍺2 human chondrocytes specifically for Gabarapl1 (left) and Beclin-1 (right) shown as untreated (CTRL) or 1 h of CGS. Both proteins are demonstrated by green fluorescence. Development of punctate autophagy initiation complexes can be observed after 1 h. These are 40 × hpf views of representative cells repeated in 3 separate experiments. (**C**) Western blot for Gabarapl1 protein levels in total cellular lysates from TC28a2 cells treated ± CGS21680 (1.45 ± 0.10 vs. 1.0 ± 0.25, n = 3–4 per group, *p* < 0.05, Student’s T-test).

To assess the effect of A2AR ligation on known FoxO-regulated autophagy proteins and their effect on cartilage autophagy, we evaluated levels of Gabarapl1 and Beclin-1. A2AR ligation led to significant increase in gene expression of both Gabarapl1 and Beclin-1 as assessed by RT-PCR (Fig. 3A). Gabarapl1 is an autophagosome-associated LC3-like *Atg8* homologue involved in both general autophagy and more specifically in mitophagy. Protein levels of Gabarapl1 are clearly increased in a punctate distribution by IF after A2AR stimulation in vitro (Fig. 3B, left column). Similarly, A2AR stimulation increases activity of the central pro-autophagy regulating kinase Beclin-1 which leads to formation of autophagy initiation complexes that can be observed by IF as punctate regions of predominantly perinuclear fluorescence compared to faint, diffuse fluorescence pattern in untreated cells (Fig. 3B, right column). Gabarapl1 protein levels were increased, as measured by western blot, in correlation with the IF results (Fig. 3C). These findings support the p62 result indicating A2AR stimulation promotes autophagic flux in chondrocytes in vitro and suggest involvement of FoxO transcription factors increasing expression of known target pro-autphagy proteins Gabarapl1 and Beclin-1.

### A2AR stimulation leads to an in vitro decrease in apoptosis-associated proteins p53 and caspase 3 and increase in chondrocyte cell viability

**Figure 4:**
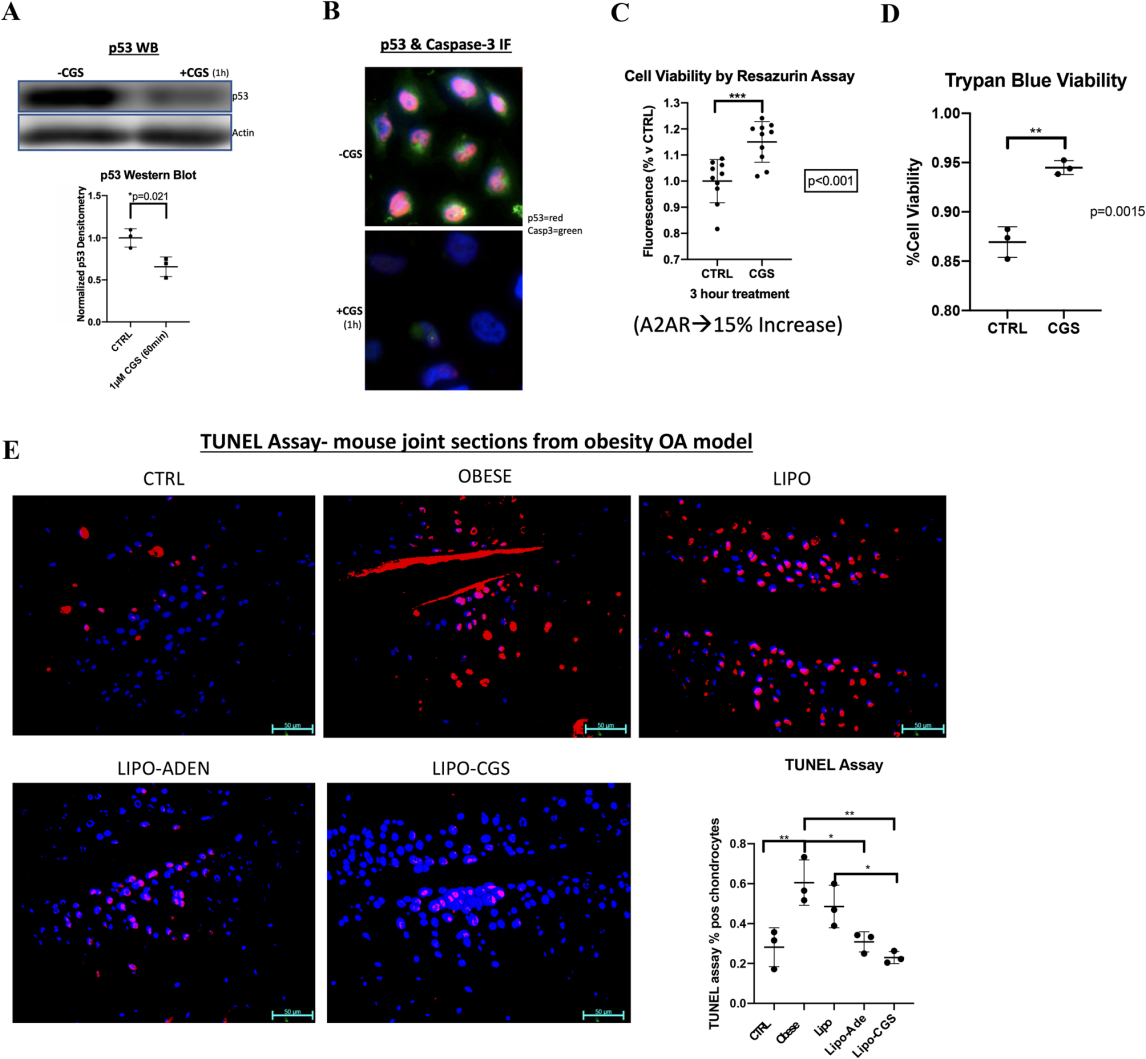
The increased autophagy observed with A2AR stimulation is also associated with decrease in apoptosis markers in TC28⍺2 human chondrocytes. (**A**) Western blot of total protein extract in TC28⍺2 human chondrocytes treated with or without 1 µM CGS for 1 h measuring p53 and loading control beta-actin. Calculation of p53/Actin densitometry in separate experiments from TC28a cells treated with or without CGS21680 vs. CTRL (0.69 ± 0.072 vs. 1.0 ± 0.067, n = 3, *p* < 0.05)). (**B**) CGS21680 treatment of TC28a2 cells by IF shows decrease in p53 (red) after 1 h 1 µM CGS21680 treatment (+ CGS) compared to control (-CGS). (**C**) Resazurin assay for cell metabolic viability in TC28⍺2 compares 1-h CGS21680 treated versus untreated TC28a2 chonodrocytes. This assay demonstrated a significant increase in metabolic chondrocyte function as a correlative cell viability marker based on cellular metabolic health; samples included n = 10 per group. (**E**) We additionally measured cell viability by the traditional Trypan Blue method and observed a significant increase in A2AR associated viability. In vitro viability assessment in TC28a2 cells treated with A2AR agonism using 1 µM CGS21680 for 3 h vs. control untreated. (**D**) In vitro viability assessment in TC28a2 cells treated with A2AR agonism using 1 µM CGS21680 for 3 h vs. control untreated. Cell viability calculations reported CGS vs. CTRL (94% ± 0.7% vs. 87% ± 1.6%, *p* = 0.0015, n = 3). (**E**) TUNEL assay for in vivo apoptosis was performed and analyzed via 1-way ANOVA was performed assessing individual cell thresholded fluorescence to calculate the percent of IHC 40 × HPF in 3 mice per group by counting medial compartment intra-articular chondrocyte cell fluorescence. Each image is in anatomic alignment with the femur on the top and the tibia on the bottom. Mean percent and standard deviation for the 5 *in* vivo groups are recorded in order by non-obese CTRL, obese CTRL, liposome only joint injection, adenosine injection, and A2AR agonist injection (28% ± 9.8%, 61% ± 11%, 49% ± 11%, 31% ± 5.1%, 23% ± 3.0%); * p < 0.05, ** p < 0.01, *** p < 0.001, **** p < 0.0001, n = 3, 1-way ANOVA followed by individual comparisons using Tukey multiple comparison tests).

OA is associated with a reduced number of articular chondrocytes and an increase in chondrocyte apoptosis. Although autophagy generally enhances cell survival, there are occasional circumstances in which autophagy can lead to apoptosis^24,27^. We therefore assessed the effect of A2AR stimulation on the central apoptosis mediators p53 and caspase 3 in TC28α2 chondrocytes in vitro. We found a significant decrease in p53 levels by western blotting in total protein extracts upon addition of 1 µM CGS21680 for an hour (Fig. 4A). Furthermore, there was an IF-visible decrease in both p53 and caspase-3 an hour after treated with the A2AR agonist in vitro (Fig. 4B). Lastly, there was a significant increase of 15% fluorescence in A2AR-agonist treated TC28a2 cells (1 µM for 3 h), indicating improvement in viability, measured by resazurin assay cell viability (Fig. 4C). Trypan blue staining for cell viability also demonstrated increased viability using the same culture conditions (Fig. 4D). There was also a trend toward a significant decrease in apoptosis in TC28a2 cells under the same treatment conditions as above, as assessed by cleaved Parp-1 (Fig. S6). This in vitro decrease in apoptosis-associated markers and improved cell viability is consistent with the fact that A2AR activation likely enhances chondrocyte survival and cell function. It is also indicative that the autophagy associated with A2AR signaling more likely enhances chondrocyte cell survival without any apparent activation of autophagy-dependent apoptosis.

When studied in vivo, a TUNEL apoptosis assay analysis of joint sections from normal mice and mice with obesity-induced OA showed a significantly higher percentage of apoptotic chondrocytes in the obese untreated OA mice compared to the normal weight mice and that this increase was reduced to near control, unaffected levels with liposomal-adenosine injections and liposomal-CGS21680 injections (Fig. 4E). Injections with empty liposomes alone also reduced apoptosis, as has been observed previously^25^, although the reduction was not nearly as marked as that induced by liposomes containing either adenosine or CGS21680. Thus our results from in vitro and in vivo studies demonstrate that A2AR signaling reduces chondrocyte apoptosis in the setting of OA.

### In vivo A2AR stimulation increases active cellular FoxO1and FoxO3 level and nuclear localization and restores autophagy in a murine obesity-induced OA model

**Figure 5:**
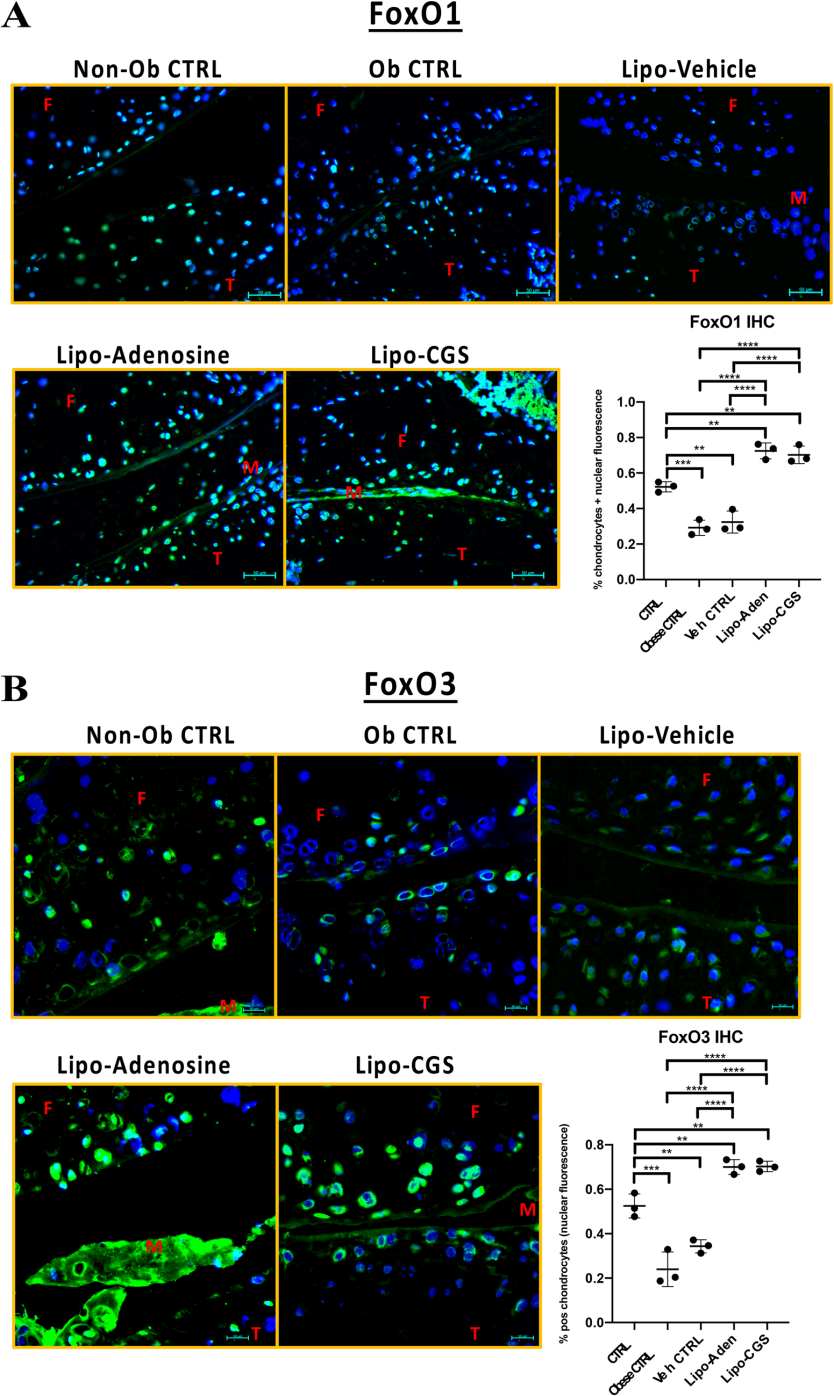
Obese mice treated with liposomal-CGS or adenosine injections exhibited elevated levels of total FoxO1 and FoxO3 with increased nuclear localization in both and reduced levels of inactive phospho-FoxO1 compared to obese controls. (**A**) 40 × hpf magnified joint sections stained for active FoxO1 (green fluorescence) using IF/IHC in the obesity mouse model of arthritis representative from 3 mice per group. A 1-way ANOVA (*p* < 0.0001) was performed assessing individual cell thresholded fluorescence to calculate the percent of IHC 40 × HPF in 3 mice per group by counting medial compartment intra-articular chondrocyte cell fluorescence. Mean percent and standard deviation for the 5 *in* vivo groups are recorded in order by non-obese CTRL, obese CTRL, liposome only joint injection, adenosine injection, and A2AR agonist injection (52% ± 2.9%, 29% ± 4.3%, 32% ± 6.2%, 72% ± 4.5%, 70% ± 5.0%; * p < 0.05, ** p < 0.01, *** p < 0.001, **** p < 0.0001, n = 3, 1-way ANOVA followed by individual comparisons using Tukey multiple comparison tests). (**B**) 40 × hpf magnified joint sections stained for active FoxO3 (green fluorescence) using IF/IHC in the obesity mouse model of arthritis representative from 3 mice per group. A 1-way ANOVA (*p* < 0.0001) was performed assessing individual cells thresholded by level/location of fluorescence was employed to calculate the percent of fluorescence per cell in IHC sections as measured from a representative 40 × HPF for sections obtained from each mice in each treatment group. Mean percent and standard deviation for the 5 *in* vivo groups are recorded in order by non-obese CTRL, obese CTRL, liposome only joint injection, adenosine injection, and A2AR agonist injection (52% ± 5.4% ± 24% ± 7.8%, 34% ± 3.0%, 70% ± 3.3%, 70% ± 2.3%; * p < 0.05, ** p < 0.01, *** p < 0.001, **** p < 0.0001, n = 3, 1-way ANOVA followed by individual comparisons using Tukey multiple comparison tests). Figure labels: F is femur, T is tibia, M is meniscus.

To determine the applicability of the in vitro demonstration that A2AR stimulation activates FoxO function and autophagy, we performed IHC to examine joint sections from obese mice that were untreated or were given intra-articular injections of liposome alone, lipo-adenosine, or lipo-CGS21680 and assessed FoxO and autophagy proteins. IHC in the lipo-adenosine and lipo-CGS21680 treated mice displayed higher levels and nuclear localization for both FoxO1 and FoxO3 with relatively decreased inactive phospho-FoxO1/3 indicating the majority of the total FoxO1/3 is active *in* vivo when compared to the obese or vehicle-only controls (Fig. 5). The increased levels of nuclear FoxO1/3 were assessed by 40 × hpf in 3 different mice and statistical calculations comparing the groups are provided (Fig. 5). Of note, we frequently observed increased background fluorescence especially in the obese mice where joint damage was greater and thus a rabbit IgG isotype control was performed for all treatment conditions and controls (Fig. S8). Similar analysis for corresponding phospho-FoxO1 and phospho-FoxO3 and showed a general decrease in nuclear and cytosolic fluorescence in the adenosine or CGS21680 injected joints by IHC (Fig. S9). Note that CGS21680 leads to relatively elevated cytoplasmic phospho-FoxO1 and phospho-FoxO3 compared to adenosine treatment. Together, these findings are most consistent with the finding that the majority of FoxO1 and FoxO3 in the joints treated with liposomal adenosine or CGS21680 were in their active form. Hence, A2AR stimulation promotes FoxO1 and FoxO3 activation and nuclear localization in vivo similar to the effects observed in vitro.

**Figure 6:**
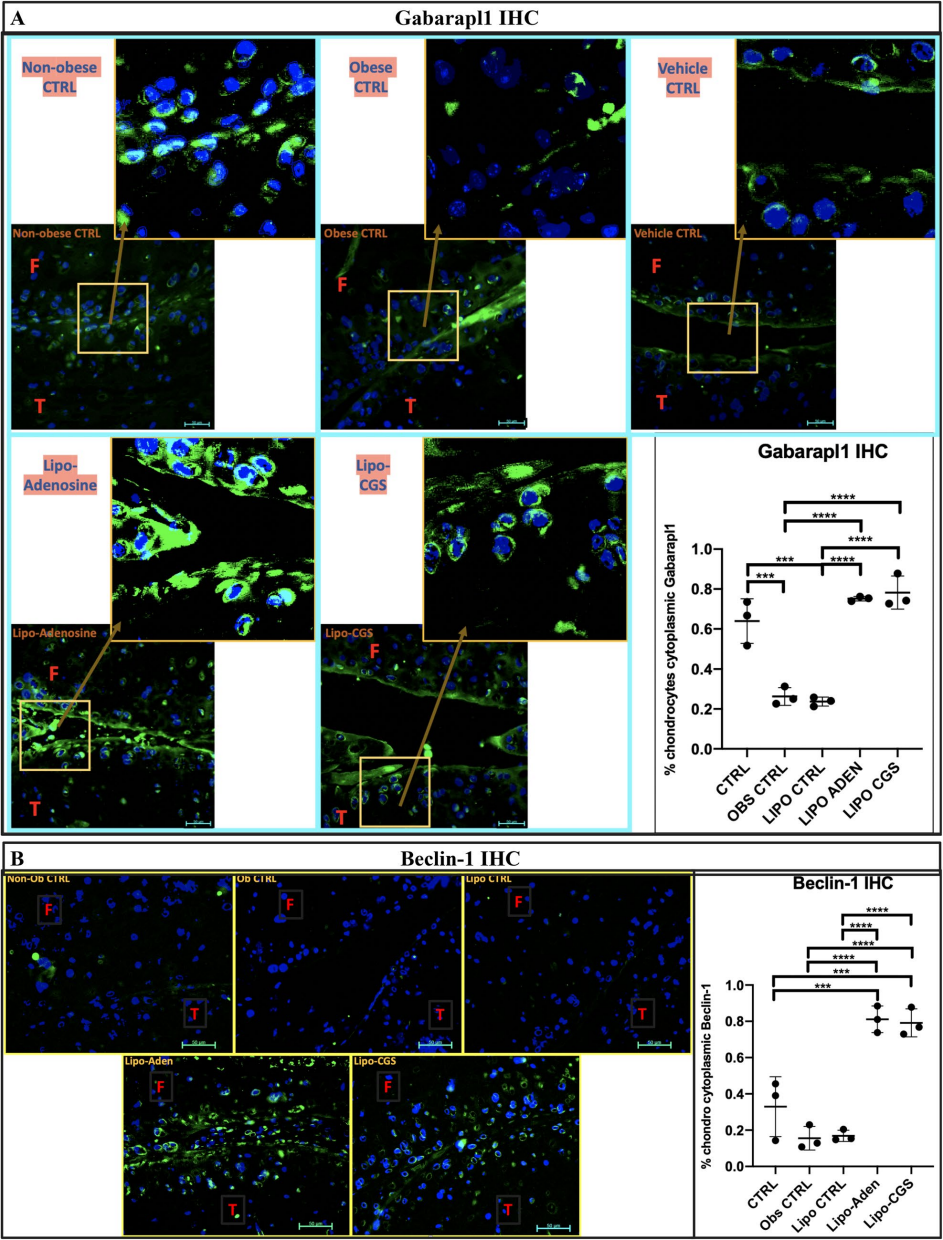
Autophagic flux is increased *in* vivo with A2AR stimulation as demonstrated for the proteins identified in vitro including Gabarapl1 and Beclin-1. (**A**) 40 × magnified joint sections (with further magnified view in box provided) stained for Gabarapl1 (green fluorescence) using IF/IHC in the obesity mouse model of arthritis. These are representative 40 × hpf images from n = 3 mice. At the bottom right, there is a 1-way ANOVA was performed assessing individual cell thresholded fluorescence to calculate the percent of IHC 40 × HPF in 3 mice per group by counting medial compartment intra-articular chondrocyte cell fluorescence. Mean percent and standard deviation for the 5 *in* vivo groups are recorded in order by non-obese CTRL, obese CTRL, liposome only joint injection, adenosine injection, and A2AR agonist injection (64% ± 11%, 26% ± 4.5%, 24% ± 2.3%, 75% ± 1.1%, 78% ± 8.3%; * p < 0.05, ** p < 0.01, *** p < 0.001, **** p < 0.0001, n = 3 per group, 1-way ANOVA followed by individual comparisons using Tukey multiple comparison tests). (**B**) 40 × magnified joint sections stained for Beclin-1 (green fluorescence) using IF/IHC in the obesity mouse model of arthritis. These are representative 40 × hpf images from n = 3 mice. Graphical analysis of the Beclin-1 IHC analysis by 1-way ANOVA using ImageJ analysis to equally threshold percentage of cells with cytoplasmic fluorescence is provided in the bottom right corner (*p < 0.05, ** p < 0.01, *** p < 0.001, **** p < 0.0001). Figure labels: F is femur, T is tibia, M is meniscus.

Diminished autophagy, which is observed in OA cartilage, can occur as a result of decreased FoxO-mediated gene expression. Therefore, similar to our in vitro analysis, we evaluated FoxO induced autophagy markers Gabarapl1 and Beclin-1 in the obesity-induced mouse OA model treated with or without liposomal injections of adenosine or CGS21680. Gabarapl1 increases cytoplasmically throughout the cartilage but is especially elevated superficially in a somewhat punctate manner (Fig. 6A). Additionally, Beclin-1 levels are greater in articular chondrocytes in the treated obese mice compared to the obese control mice and (Fig. 6B). There is very little Beclin-1 in the obese mouse controls (obese ± liposome) and also in the norma weight control. Levels of p62 were moderately increased in the obese untreated or vehicle treated obese controls compared to normal weight, control, mice and also to the mice injected with lipo-adenosine or lipo-CGS21680 (Fig. S10). Taken together these findings are consistent with the hypothesis that intra-articular treatment of OA cartilage with liposomal adenosine or CGS21680 restores autophagy in OA chondrocytes in this OA mouse model in vivo.

## Discussion

Decreased autophagy is observed in OA and aging cartilage; conversely, increasing autophagy promotes cartilage homeostasis^4,28^. We have previously reported that intraThe results from this study provide a mechanism by which A2AR activation increases chondrocyte autophagy through a pathway involving activation of the FoxO transcription factors. This pathway among others likely contributes to the significant chondroprotective effect that our lab has previously demonstrated in a rat model of PTOA treated with intra-articular adenosine that was reversible with inhibition of the A2AR^24^. It also provides an explanation for the observed phenotype in A2AR knockout mice characterized by decrease in the normal cartilage matrix constituents collagen and glycosaminoglycans and increase in metalloproteinase genes leading to chondrocyte hypertrophy and OA^29^.

Our experimental approach in these experiments was designed to evaluate the effect of A2AR signaling on the key transcription factors FoxO1 and FoxO3, previously shown by Matsuzaki et al. to be critical for maintenance of cartilage homeostasis by upregulating chondrocyte autophagy and improving oxidative stress response^18^. We found evidence for both in vitro and in vivo A2AR activity leading to enhanced levels and nuclear translocation of the FoxO proteins with an increase in activity in autophagic flux. Moreover, we found an in vitro decrease in p53, a well known mediator of cartilage cellular senescence and apoptosis depending on the cellular context via a Sirt1-p53-p21 pathway^30^. This was accompanied by evidence for increased cellular and metabolic viability as well as decreased apoptosis by TUNEL assay in obesity-induced arthritis. It is notable that the TC28a2 cells are a human cell line, they proved useful in deciphering alterations in the FoxO and autophagy markers assessed and data that has been recently published from our lab indicates a strong correlation between homeostatic changes in human primary chondrocytes and TC28a2 chondrocytes^31^. Moreover, the effects observed in this human cell line are similar to those found in murine chondrocytes in vivo.

The FoxO transcription factors are a set of evolutionarily conserved proteins that include ubiquitously expressed FoxO1, FoxO3, and FoxO4, as well as FoxO6 expressed in the brain^20^. FOXOs are transcription factors that induce expression of genes that regulate cellular aging, homeostasis, response to oxidative stress, autophagy, protein quality control, cell cycle arrest, differentiation, DNA repair, organism longevity, immune response, metabolism, and apoptosis or cell survival depending on the cell conditions^21,32^. Evaluation of healthy human cartilage demonstrated relative increase in FoxO1 and FoxO3 but not FoxO4 proteins in the superficial and mid cartilage zones and decrease in phosphorylated, inactive FoxO1 and 3^22^. A recent RNA sequencing study evaluating transcriptional networks in human OA showed downregulation of the FoxO signaling pathway in OA patients^33^. Importantly, while FoxO1 and FoxO3 are often regulated similarly in these experiments, as we observed, FoxO3 may also exit the nucleus to the mitochondria where FoxO3 plays a role in reduction of excessive ROS and mitophagy by specifically targeting depolarized mitochondria and avoiding normally functioning mitochondria^34^. Hence, this alternate route of trafficking would lead to greater cytoplasmic localization of FoxO3 compared to FoxO1. Furthermore, it is possible that adenosine receptor specific differences between receptor non-specific adenosine and A2AR specific treatment with CGS21680 may have slightly different effects by involvement of other adenosine receptors. For example, the low affinity A2BR adenosine receptor that is upregulated in hypoxic conditions may play a similar role as compared to selective activation of A2ARs^35,36^. Nonetheless, A2AR signaling is likely the predominant physiologic homeostatic mechanism in cartilage, given our laboratory’s previous demonstration that A2BR blockade does not affect the capacity of liposomal adenosine to reduce osteoarthritic changes in cartilage as well as the higher affinity of A2AR than A2BR for adenosine (nM vs. μM) in the setting of generally low physiologic levels of extracellular adenosine^24,35^.

The obese mouse OA model employed in this study as mentioned develop early, severe arthritis and are especially prone to metabolic defects, inflammation, and mitochondrial dysfunction^25,31^. It is also important to understand that while FoxO1 and FoxO3 often play similar roles, they do have specific functions. FoxO3 often accompanies FoxO1 into the nucleus but can also be translocated to the mitochondria to exert oxidative protection, mitophagy, and mitochondrial biogenesis programs, usually in conjunction with Sirt3^34^. This may account for slight differences observed with respect to timing of protein increase and localization. Importantly, we have recently demonstrated that A2AR activation leads to improved mitochondrial function, mitophagy, biogenesis and proper cristae appearance^31^. Further assessing the connection between FoxO-mediated autophagy, upstream signaling potentially via AMPK and Sirt1, and the effect on chondrocyte mitochondria is an important future direction.

As mentioned initially, the specific autophagosome proteins Gabarapl1 and p62 have some unique qualities that may lead to chondrocyte homeostasis, mitochondrial quality control, and prevention of cellular senescence. We were specifically interested in Gabarapl1 because it is important for mitochondrial quality control and it was identified by our lab to be significantly increased in joints of rats with post-traumatic OA injected with liposomally-attached adenosine A2AR agonist CGS21680 (unpublished RNA sequencing data)^15^. Similarly, its cartilage expression was significantly decreased in FoxO1/3/4 triple knockout mice as well as both FoxO1 and FoxO3 single knockout mice^18^. The same group showed Gabarapl1 expression was increased in cultured human chondrocytes and immature murine articular chondrocytes (IMACs) with each cell type overexpressing FoxO1. In fact, there was an especially high fold change in Gabarapl1 in the IMACs compared to other autophagy genes displayed^18^. With respect to chondrocyte senescence, p62 function has been shown to be essential in protecting cells from NFκB activated inflammation and senescence, two processes increased in OA^37^.

With respect to possible regulation of FoxO levels, localization, and activity, although AKT is a major inhibitory regulator of FoxOs by phosphorylation at three sites, the inhibition of AKT by A2AR signaling is unlikely to explain the observed FOXO changes given the fact that A2AR actually leads to early activation of AKT observed in osteoblasts^38^, fibroblasts^39^, and recently chondrocytes^25^. Furthermore, there was an A2AR specific effect with moderately higher cytoplasmic phospho-FoxO1 and phospho-FoxO3 in mice treated with liposomal-CGS21680 compared to liposomal-adenosine, which may result from an A2AR-specific known elevation in active AKT in chondrocytes and other cell types^38,40^. Analysis of how these pathways coexist to induce homeostasis will be an interesting future topic of study. Other upstream kinases that may regulate the FoxO proteins are abundant. Negative regulators include Nemo-like kinase (NLK), serum and glucocorticoid-inducible kinase (SGK), dual-specificity tyrosine-phosphorylated and regulated kinase (DYRK), Ikappa B kinase (IKKβ), ERK1/2, as well as cyclins CDK1 and CDK2^41–47^. Positive kinase regulators include AMPK, JNK (responsive to increase in cellular ROS), macrophage stimulating 1 (MST1), casein kinase 1 (CK1), STAT3, and the p38 mitogen activated protein kinases^20,48–54^.

AMPK, which is decreased in OA and aging, is of interest with respect to chondrocyte autophagy given it promotes FoxO1 and FoxO3 activation, inhibits mTOR, and it appears to be chondroprotective in response to starvation, ROS, and hypoxia^55^. AMPK is activated by an upstream kinase, Liver Kinase B1 (LKB1), which has been shown to be essential to maintenance of healthy cartilage generally by phosphorylating and activating AMPK^56–58^. AMPK can activate the NAD-dependent Sirt1 deacetylase, which is also a pro-longevity and autophagy protein that increases cartilage homeostasis and cell survival via targeted deacetylation of key target proteins^59^. Sirt1 activates FoxO1 and FoxO3 by deacetylation, which increases nuclear retention and DNA binding allowing for more efficient transcription. Moreover, deacetylated FoxO1 and FoxO3 have certain alterations in gene targets compared to the acetylated FoxOs. Deacetylated FoxO1 and FoxO3 lead to a pro-survival, anti-apoptotic, pro-autophagy stress responsive pathway by upregulated autophagy genes and oxidative stress response genes^60^. Other potential chondroprotective mechanisms of the AMPK-Sirt1 axis aside from FoxO activation include Sirt1-mediated deacetylation of p53 causing its degradation^61^. Thus, while the observed decrease in p53 and caspase 3 in response to A2AR stimulation may be related to downstream autophagy-induced survival, it may also be attributable to upstream Sirt1 action on its other targets. Sirt1-associated changes would be beneficial in promoting chondrocyte homeostasis since OA is characterized by chronic low-grade inflammation, metabolic derangements often secondary to increased adiposity and diminished mitochondrial function.

**Figure 7:**
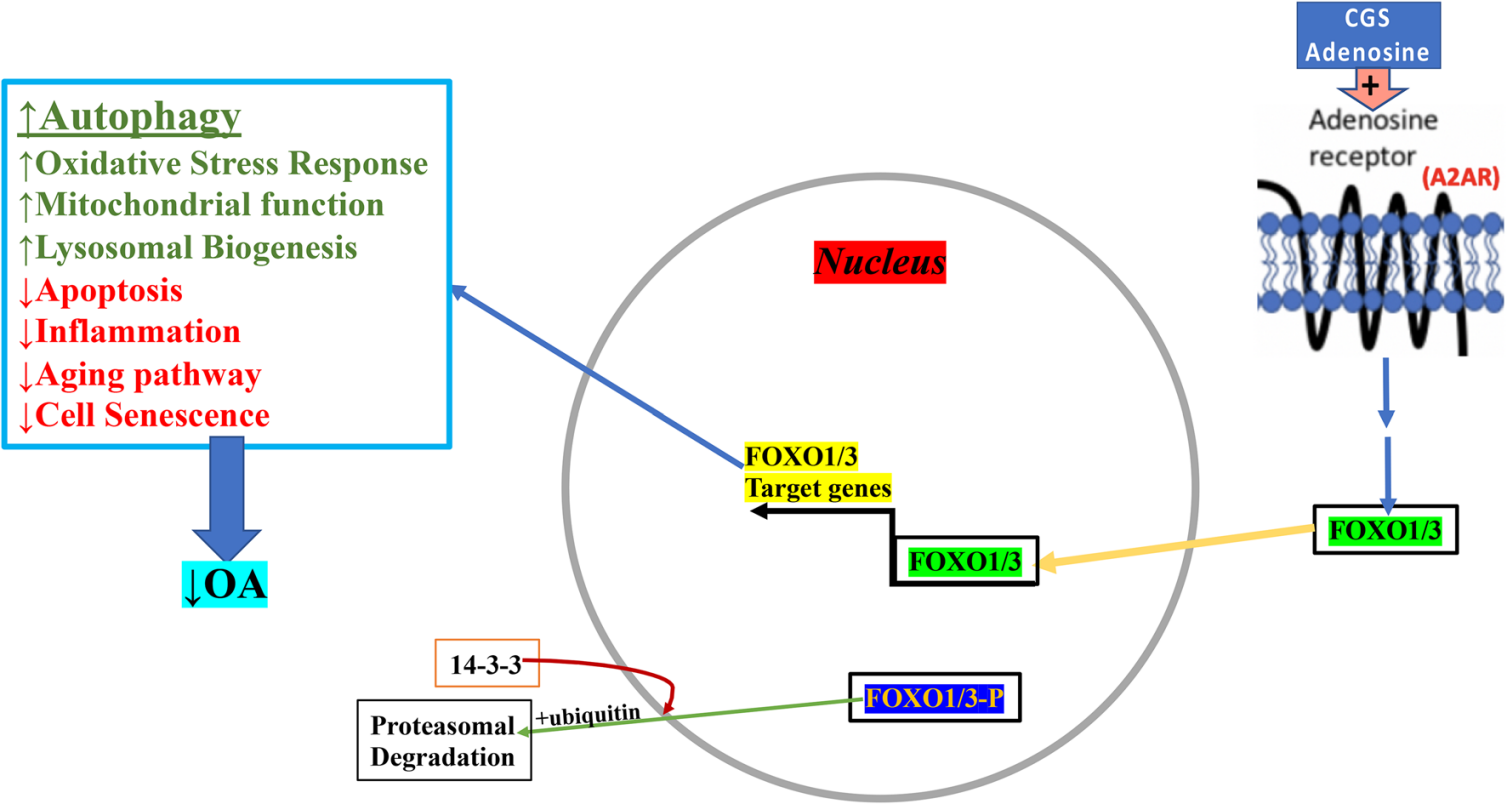
Proposed model for A2AR activation and activation of autophagy. CGS21680 or adenosine binds the A2AR, a Gs-associated GPCR which signals via cAMP to activate PKA or EPAC1/2 (not shown) leading to activation of downstream signaling culminating in nuclear transport and activation of FoxO1/3. There is also nearly simultaneous export of initially nuclearly-located inactive AKT-phosphorylated FoxO1/3 that exits the nucleus on its way to proteosomal degradation. Active FoxO1/3 are known to lead to up-regulation of autophagy genes in addition to participation in other homeostatic pathways as listed in the box on the left. These processes lead to a reduction of OA with improved health of articular cartilage.

Our results, in conjunction with our recently published studies^25,31^, demonstrate that active FoxO1/3 translocate into the chondrocyte nucleus and this translocation is associated with an increase in autophagic flux in vitro and in vivo. Together this is indicative of a potential autophagy-related homeostatic and chondroprotective mechanism mediated by A2AR signaling through the homeostatic FoxO signaling pathway leading to improvement in cartilage health (Fig. 7). The A2AR GPCR is Gs associated and hence activates cAMP as a second messenger, which activates PKA and EPAC1/2. PKA can potentially activate some of the upstream nutrient sensing kinases such as AMPK or LKB1 and it can also directly phosphorylate Sirt1 deacetylase in some cases^62,63^. Thus, we identified a novel A2AR signaling mechanism involving FoxO1/3 and propose a potential set of FoxO upstream regulators that need to be thoroughly evaluated for confirmation.

## Methods

### Materials

CGS21680 (A2AR agonist) and ZM241385 (A2AR antagonist) was obtained from TOCRIS (MI, USA). Hydroxychloroquine (HCQ) was obtained from Thermo Fisher Scientific/Acros Organics (NJ, USA). Antibodies: Ms-SQSTM1/p62 ab56416, Rb-Beclin-1 ab62557, Rb-Gabarapl1 ab86497, Rb-FOXO1A (ab 39,670), Rb-Phospho-FOXO1 (S256) (ab131339), Rb-FOXO3A (ab12162), Rb-Phospho-FOXO3 (S253) (ab47285), Ms-nuclear matrix protein p84 (ab487), and Apoptosis Western Blot Cocktail pro/p17-caspase-3, cleaved PARP1, muscle actin (ab136812) were purchased from ABCAM (MA, USA); Rb-Caspase 3 sc-7148 was purchased from SantaCruz (CA, USA). Ms-p53 antibody was purchased from Cell Signaling (Danvers, MA, USA). Paraformaldehyde (PFA) 16% was obtained from Electron Microscopy Sciences (PA, USA). RIPA buffer, EDTA, bovine serum albumin, Anti-Rabbit IgG–FITC antibody, Anti-Rabbit IgG–TRITC antibody, ethanol, glycerol, and adenosine were purchased from Sigma-Aldrich (MO, USA). Alexa Fluor 555 phalloidin, DMEM, penicillin–streptomycin and fetal bovine serum were purchased from Life technology (NY, USA).

### Animals^24,64,65^

Mice utilized in this study are the subject of a separate report^65^. The mice were kept under regular lighting conditions (12 h light/dark cycles) and given food and water ad libitum. As described by our lab previously^64,65^, 12 week old C57/Bl6 mice (Taconic Bioscience Laboratories, Hudson, NY, USA), were fed a high fat diet (60% fat, Research Diets D12492i—Research Diets Inc., NJ-USA) for 12 weeks before and during intraarticular treatment with saline or liposome with and without CGS21680 or adenosine with 10 µl injections every 10 days for 6 injections. Mice were euthanized by CO_2_ narcosis. Knee joints were fixed and embedded in paraffin. All protocols were approved by the New York University School of Medicine Institutional Animal Care and Use Committee.

### Liposomal CGS12680 and adenosine treatment^24,64,65^

Liposomes were prepared fresh the day of injection. Ethanol was added to soybean oil containing adenosine or CGS21680. The lipid phase containing phosphatidyl choline and cholesterol (1:0.5 by molar ratio) was added to the previous solution and emulsified at 15,000 rpm for 10 min. Saline along with glycerin was then added to the lipid phase and was homogenized at 15,000 rpm for 20 min followed by sonication for 1 min at 100% duty cycle. Adenosine encapsulation in liposome was measured after 1, 2, 24, 48 h incubation at 37 °C. Liposomes were incubated in PBS and then collected at the different time points. Liposomes were centrifuged at 23000 g for 1 h at 4 °C. Supernatant was removed and the liposome pellet was resuspended in a saline solution containing 0.5% Triton-X100. Adenosine concentration in the remaining intact liposomes was quantified by High Performance Liquid Chromatography^24,64,65^.

### Histology and immunohistochemistry^24,64,65^

Hindlimbs from mice were cleaned of soft tissue, placed into 4% PFA for 48 h, and decalcified in 10% EDTA for 4 weeks. Paraffin-embedded histological Sects. (5 mm) were cut, mounted and prepared for analysis. Joint sections were deparaffinized by xylene and re-hydrated in decreasing ethanol concentrations. Sections were depleted of endogenous peroxidase activity with 3% H_2_O_2_ in methanol, then blocked with PBST containing bovine serum albumin (1%) and FBS (5%) for 60 min. Sections were incubated overnight with specific primary antibodies at manufacturer-specified dilutions. After rinsing with phosphate-buffered saline (PBS), horseradish peroxidase (HRP)-conjugated secondary antibody was applied and stained with diaminobenzidine (DAB) kit.

### Chondrocyte cell culture^24,64,65^

TC28α2 chondrocyte cells were maintained in Dulbecco’s Modified Eagle’s Medium (DMEM) (MilliporeSigma, Burlington, MA, USA) supplemented with 10% fetal bovine serum (FBS) and 1% Penicillin–Streptomycin. They were incubated under normal culture conditions at 37 °C supplemented with 5% CO_2_. In certain experiments, cells were subjected to serum starvation conditions in DMEM with 1% FBS for the identified time. Cells were treated with 1 µM adenosine A2A receptor (A2AR) agonist CGS21680 for indicated time points (between 30 min and 3 h). In certain cases, A2AR antagonist ZM241385 at 1 µM was added 15 min prior to addition of CGS21680. Autophagy inhibitors hydroxychloroquine (HCQ) at 25 µM and 3-methyladenine (3-MA) at 5 mM were added approximately 16 h prior to treatment of cells with CGS21680. TC28α2 chondrocyte confluence was approximately 80–90% prior to protein extraction for western blotting and 70–80% prior to visualization by immunofluorescence.

### Protein extraction and western blotting^24,64,65^

After cell treatment as above, cells were collected and lysed with RIPA buffer containing protease and phosphatase inhibitors. Alternatively, nuclear and cytoplasmic protein extracts were isolated using the NE-PER nuclear and cytoplasmic extraction reagent kit (Thermo Scientific, Waltham, MA, USA) in accordance with the manufacturer’s instructions. Protein concentration was assessed with the BCA kit (Thermo Scientific, Waltham, MA, USA) and sample absorbance at 750 nm was used to estimate protein concentration using the Synergy H1 Hybrid Multi-Mode Microplate Reader (Biotek, Agilent, Winooski, VT, USA) and data analysis was performed using SoftMax Pro Version 7 (Molecular Devices, Sunnyvale, CA, USA). Western blotting was performed by electrophoresing 20 µg of protein through 8–16% polyacrylamide Mini-PROTEAN pre-cast protein gradient gels (Bio-Rad, Hercules, CA, USA) followed by transfer of proteins to nitrocellulose membranes. Nitrocellulose membranes were incubated overnight at 4 °C with the specific primary antibody (1:1000), and after washing, incubated with goat anti-rabbit IRDye 800 CW and goat anti-mouse IRDye 680 RD (1:5000). Membranes were scanned with LI-COR Odyssey equipment and the intensities of the protein bands were quantified by densitometric analysis using Image Studio 2.0.38 software.

### Immunofluorescence^24,64,65^

TC28α2 cells were plated in 8-well chamber slides, treated as mentioned above, and then washed with cold PBS and fixed with 4% paraformaldehyde. Cells were permeabilized with 0.25% Triton in PBS for 10 min. After 3 washes for 5 min each, a blocking solution of 1% BSA in PBST was added to the cells for 1 h. Cells were incubated with each specific primary antibody overnight at manufacture specified dilutions. After washing with PBS, cells were incubated with the secondary antibodies FITC conjugate (1:100 in PBST) and/or TRIC-labelled Phalloidin (1:100 in PBST) for 1 h at room temperature. After washing, a cover slide was applied to the slide with a mounting media containing DAPI. Immunofluorescence was revealed by Alexa Fluor 488 nm (green) and Alexa Fluor 594 nm and visualized by IF microscope and images taken by camera.

### mRNA extraction, reverse transcription, and real time PCR (RT-PCR or qPCR)

Total RNA from was extracted from TC28α2 cells using an RNeasy Mini Kit (Qiagen, Invitrogen) and QIAshredder columns (Qiagen, Invitrogen), following the manufacturer’s protocol. RNA reverse transcription was carried out using the MulV Reverse Transcriptase PCR Kit (Applied Biosystems). Upon conversion to cDNA, real time PCR reactions were performed for relative quantification of P62/SQSTM1(forward: 5′-TGCCCAGACTACGACTTGTG-3′; reverse: 5′-AGTGTCCGTGTTTCACCTTCC-3′), BECN1 (forward: 5′-ACAGTGGACAGTTTGGCACA-3′; reverse: 5′-CGGCAGCTCCTTAGATTTGT-3′), and GABARAPL1 (forward: 5′-AGGAGGACCATCCCTTTGAGT-3′; reverse: 5′-TGGCCAACAGTAAGGTCAGA-3′) as compared to control GAPDH (forward: 5′-GACATCAAGAAGGTGGTGAA-3′; reverse: 5′-TGTCATACCAGGAAATGAGC-3′). RT-PCR was performed on a Stratagene Mx3005P (Agilent Technologies) with Brilliant SYBR Green Kit QPCR Master Mix (Stratagene, Agilent Technologies), according to the manufacturer’s protocol.

### Resazurin cell viability assay^21^

TC28α2 cells at 90% confluence in 96 well plates were treated with or withot CGS21680 for 3 h. Sample fluorescence using the Synergy H1 Hybrid Multi-Mode Microplate Reader (Biotek, Agilent) and data analysis was performed using SoftMax Pro Version 7 (Molecular Devices). The resazurin assay kit (abcam, ab129732) was used to analyze cell viability by incubating each sample with 20X cell viability solution for 4 h prior to fluorometric analysis. The fluorescence resulting from reduction of resazurin to resorufin was measured with excitation and emission wavelengths of 530 and 570 nm, respectively. Cell viability was calculated as the percent increase in fluorescence of CGS21680-treated cells compared to fluorescence in untreated cells.

### Trypan blue cell viability

TC28α2 cells at 80–90% confluence in 6-well plates were treated with or without 1 µM CGS21680 for 3 h, incubated in normal cell culture conditions, trypsinized for 5 min, centrifuged at 1000 rpm for 5 min at room temperature, and resuspended in PBS with Trypan blue (Thermo Scientific, Waltham, MA, USA) and counted without sample identifiers visible. The percentage of viable cells for each sample were recorded and identities of sample were revealed.

### TUNEL assay

Individual knee joint paraffin-embedded samples were assessed in untreated normal weight mice, untreated obese mice, liposome-only injected obese mice, liposomal-adenosine treated obese micel and, liposomal-CGS21680 treated obese mice as mentioned above. TUNEL assay was performed for paraffin embedded sections for 3 mice per group as per Abcam protocol using the TUNEL Assay Kit – BrdU-Red (ab66110) (Abcam, MA, USA).

### Statistical analysis

Statistical comparisons and significance were determined using an unpaired Student’s *T*-test, one-way analysis of variance (ANOVA) followed by Tukey’s multiple comparison tests, or mixed model ANOVA for repeat-measures, as appropriate, using Prism 8 (GraphPad Software, Inc., La Jolla, CA, USA). Microsoft excel was used for creation of line graph. The results are reported as mean ± SD with p-values less than 0.05 considered statistically significant.

### Study approval

All animal protocols were approved by the New York University School of Medicine Institutional Animal Care and Use Committee. All methods were performed in accordance with New York University Medical Center guidelines and regulations.

## Supplementary information

Supplementary Figures.

## Data Availability

Supporting data for this research are available from the corresponding author upon request.
