# Supplementary material for: Adenosine A2A receptor signaling promotes FoxO associated autophagy in chondrocytes
Source: Sci Rep. 2021 Jan 13;11:968. doi: 10.1038/s41598-020-80244-x (PMC7806643; doi:10.1038/s41598-020-80244-x)
Supplement: Supplementary file 1 — Supplementary Figures. [file 41598_2020_80244_MOESM1_ESM.pdf]

SUPPLEMENTAL FIGURES:

Title: Adenosine A2A Receptor Signaling Promotes Autophagic Homeostasis Via a FoxO Transcription Factor Regulatory Pathway

Authors: Benjamin Friedman MD[1,2], Carmen Corciulo PhD[2], Cristina Castro-Rivera[2], Bruce N Cronstein MD[1,2]

Author Affiliations:
1. Department of Medicine-Division of Rheumatology-NYU School of Medicine, 550 First Avenue, New York, New York 10016, USA
2. Department of Medicine-Division of Translational Medicine-NYU School of Medicine, 550 First Avenue, New York, New York 10016, USA

Corresponding Author: Bruce Cronstein MD
Correspondence emails to bruce.cronstein@nyulangone.org

Gels from TC28a2 Nuclear Fractions- WB FoxO1

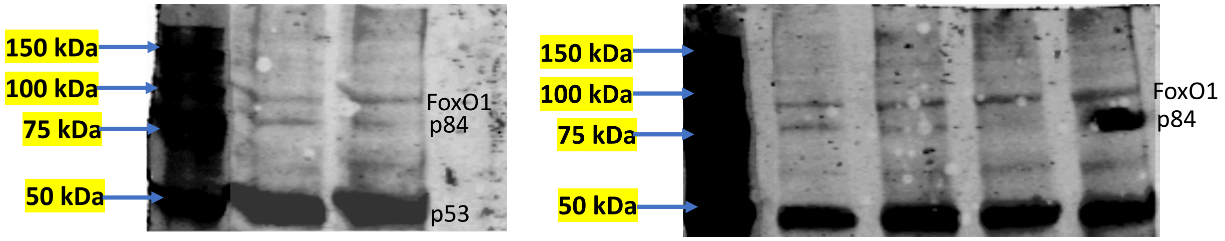

Gel from TC28a2 Nuclear Fraction- WB FoxO3

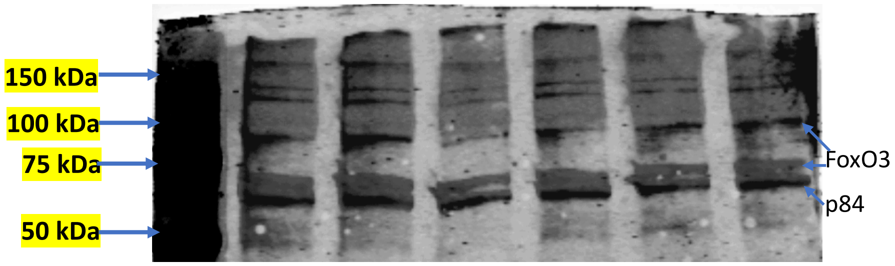

Fig S1. Western blot demonstrating relative FoxO1 and FoxO3 gels demonstrating concordance with corresponding nuclear matrix protein p84 in each lane analyzed. Western blot protein bands detected via red fluorescence (mouse primary antibody against Nuclear Matrix p84). The expected MW of this protein is approximately 84 kDa but may be found at lower weights frequently 75 kDa. Molecular weight markers are to the left adjacent to the lanes of interest. p53 (red, opposite color as FoxO1) was assessed at an earlier time point on this gel as visible on the FoxO1 blot at 30 minutes as identified though had no overlap with any of the bands of interest. The expected MW of FoxO1 is around 70 kDa however the protein is often visible as single or multiple bands at slightly higher molecular weights between 78-82 kDa in addition to the expected miolecular weight around 70-75 kDa. The predominant band size here that correlated most with FoxO1 signal was located approximately 80 kDa. FoxO3 can also have multiple bands between about 60-90 kDa as shown. Note the top 2 gels were performed in parallel but run on separate gels secondary to number of wells needed for SDS-PAGE.

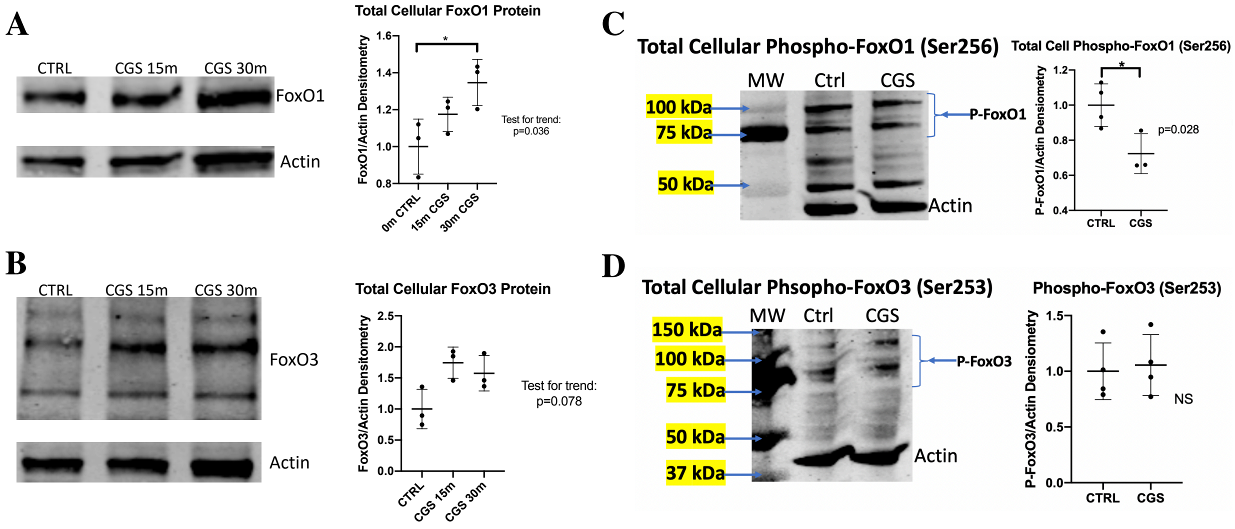

Fig S2. Total cellular FoxO1 and FoxO3 increase over time with a concomitant decrease in inactive phospho-FoxO1 in TC28a2 cells treated with CGS21680. (A) Total cellular FoxO1 western blot and 1-way ANOVA repeated measures with significant increase in trend over 0, 15, and 30 minutes. **(B)** Total cellular FoxO3 western blot and 1-way ANOVA repeated measures with significant increase in trend over 0, 15, and 30 minutes, FoxO3 here peaks earlier at 15 minutes. **(C)** Western blot of total cellular protein from TC28α2 human chondrocytes treated with or without 1μM CGS for 30m assessing inactive phospho-FoxO1 (Ser256) protein levels showing normalized levels CGS vs control of 0.72±0.1 vs 1.0±0.1 (mean ± standard deviation), *p<0.05 by unpaired t-test, n=3-4 per group. **(D)** Western blot for total cellular from TC28α2 human chondrocytes treated with or without 1μM CGS for 30m assessing inactive phospho-FoxO3 (Ser253) protein showing similar levels in CTRL and CGS21680.

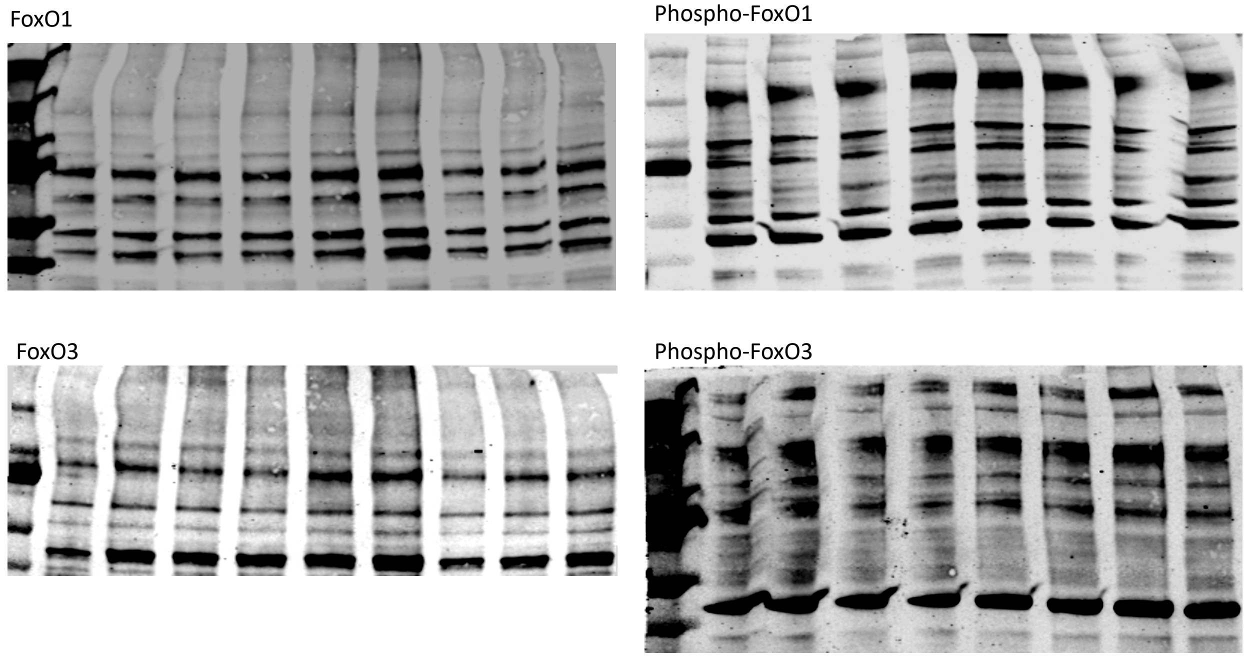

Fig S3. Western blots demonstrating total cellular FoxO1, FoxO3, phospho-FoxO1, phospho-FoxO3 with Actin loading control. FoxO1 and FoxO3 lanes from the molecular weight marker are untreated, 15 minutes CGS21680, 30 minutes CGS21680 (repeated thrice). Phospho-FoxO1 and phospho-FoxO3 lanes from the molecular weight are simply untreated and 30 minutes CGS21680 (alternating and repeating 4 times from MW markers). MW markers visible 150, 100, 75, 50, 27, 25 kDa.

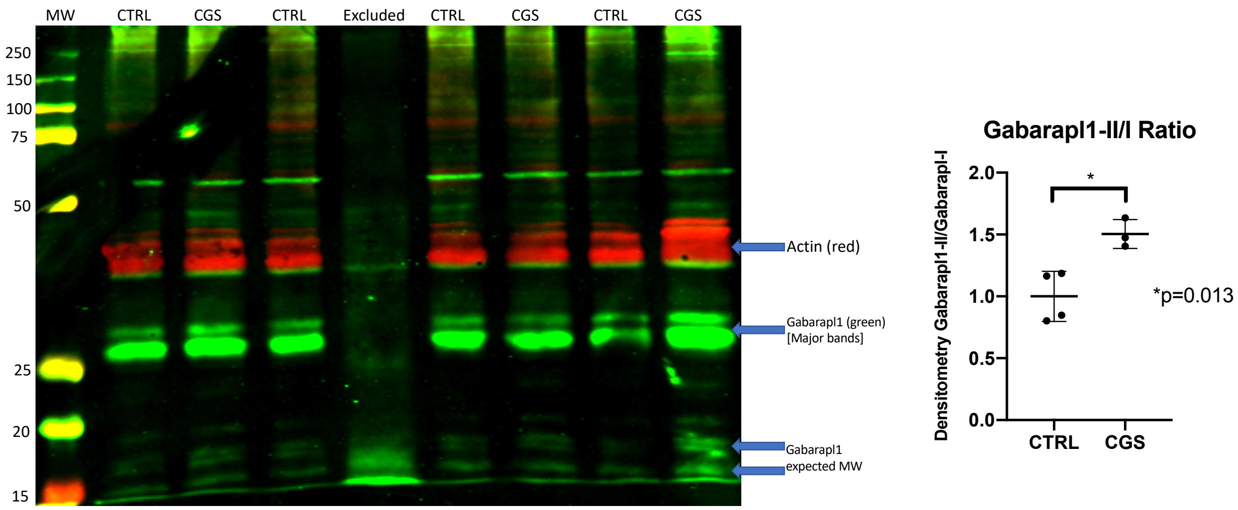

Fig S4. Color gel shows major Gabarapl1 protein bands and actin (left). Alternate analysis demonstrates increase in lipid-associated Gabarapl1 (lower MW) compared to its delipidated form (high MW) as assessed via its major bands. Note ubiquitination is a potential post-translational change for Gabarap proteins when assessing expected versus visualized molecular weights (see reference 17).

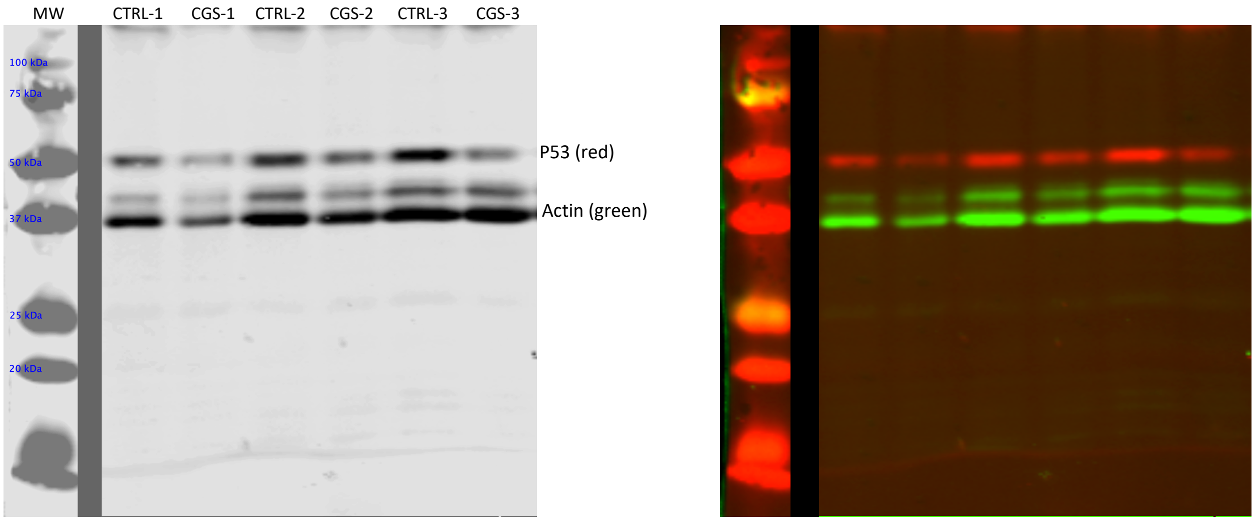

Fig S5. Western blot gel comparing p53 in TC28a2 cells treated with or without CGS21680 for 60 minutes. Overlayed black and white image (16-bit) demonstrating p53 and Actin (present as double band with predominant protein level in the lower band) is on the left. Color image on the right shows p53 in red (mouse primary antibody) and actin in green (rabbit primary antibody). The black line indicates other samples were run between the molecular weight (MW) lane and the sample lanes on the same gel with direct horizontal transposition from right to left. Note the antibodies are described in the methods section.

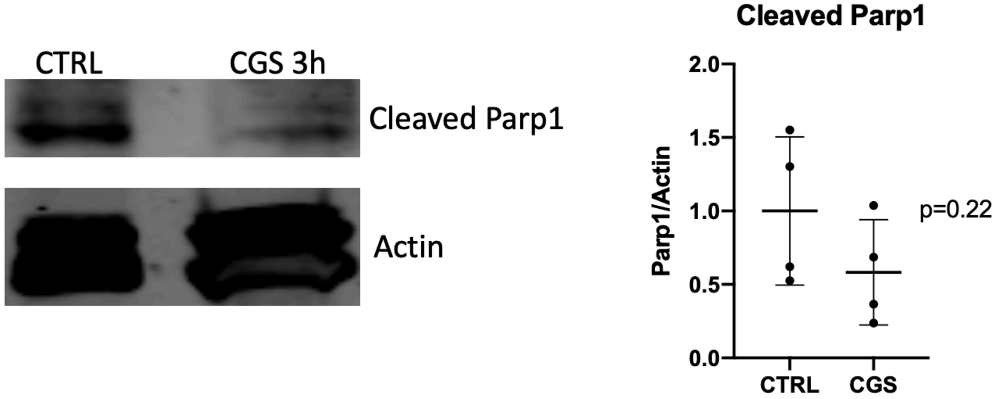

Fig S6. A2AR activation indicates a trend towards decreased apoptosis *in vitro* as assessed by Parp1 cleavage. Cleaved PARP1 representative of *in vitro* apoptosis that is trending trending lower in CGS treated versus untreated TC28a2 cells treated with 1µM CGS21680 over 3 hours. No inflammatory or oxidative stimulus was used to assess for differences in apoptosis in basal cell culture conditions (80-90% confluency). 3h CGS21680 vs untreated control (CTRL) measurement for cleaved PARP1/Actin densitometry showed mean level ± standard deviation of 0.58±0.36 vs 1.0±0.50, p=0.23, n=4, t-test.

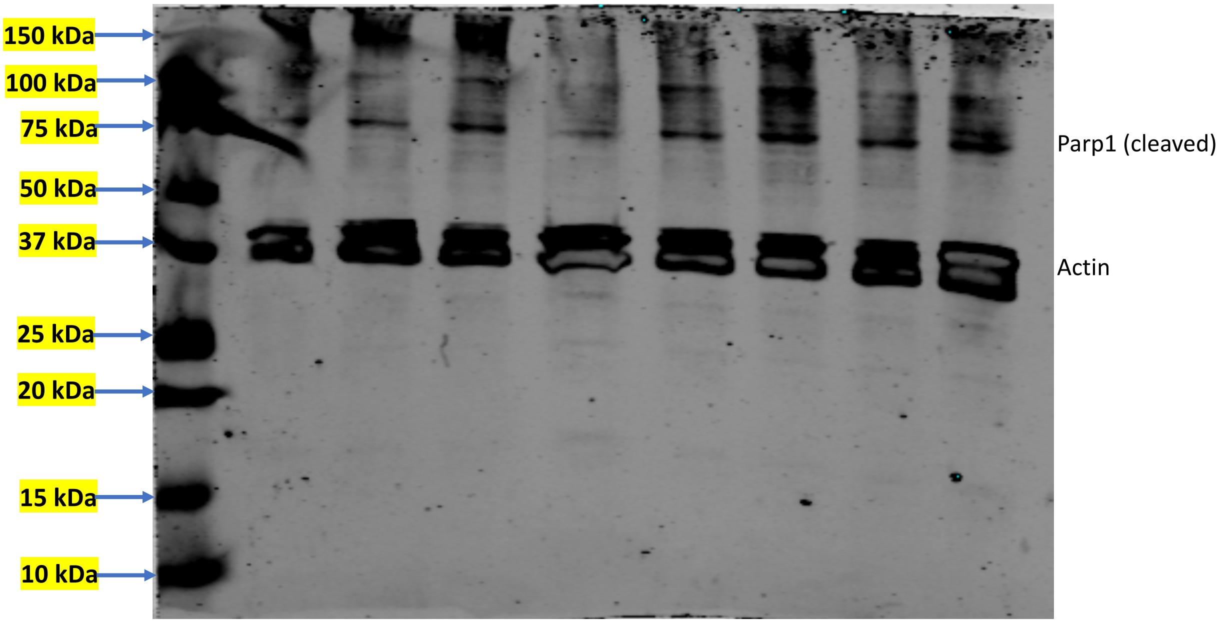

Fig S7. Full gel assessment of Parp1 cleavage analysis indicates a potential trend towards increased apoptosis in TC28a2 chondrocytes treated with CGS21680 for 3 hours compared to basal culture conditions. Alternating lanes from the molecular weight markers beginning with CTRL then CGS.

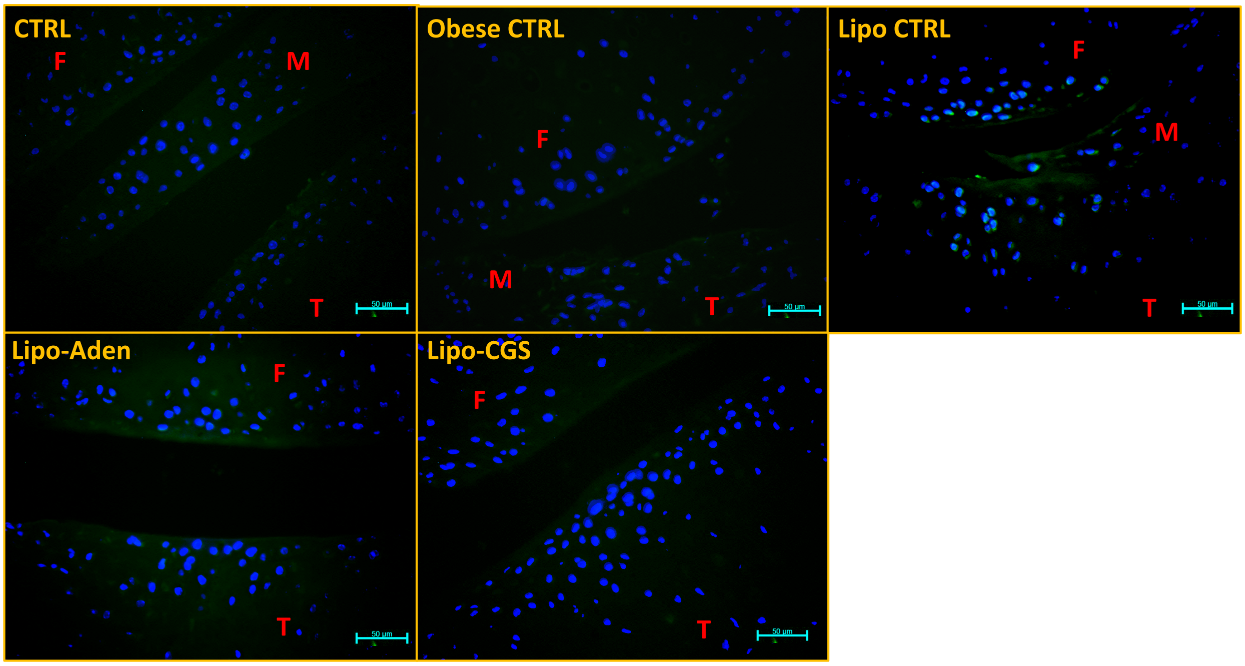

Fig S8. Immunohistochemistry rabbit IgG isotype control for green fluorescence. These are representative 40x HPF views of the same sections in the main article that involve staining with a non-specific IgG followed by the same goat anti-rabbit that would be expected to fluoresce green with non-specific staining.

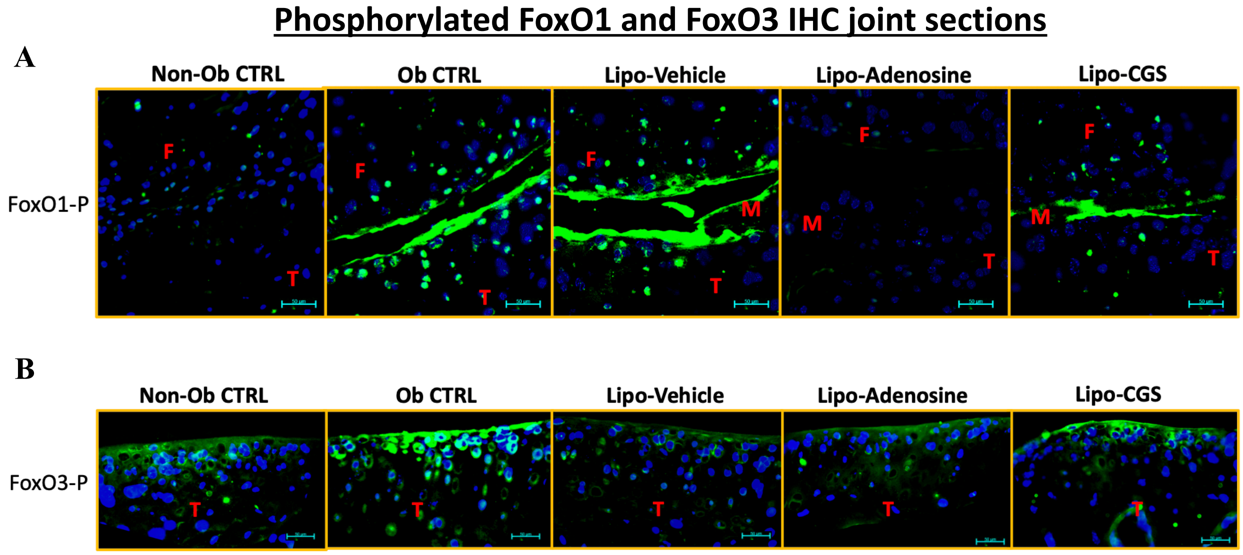

Fig S9. IHC staining for phospho-FoxO1 and phospho-FoxO3 shows a representative decrease in phospho-FoxO1 and 3 in normal control, liposomal adenosine and liposomal CGS21680. In these representative 40x hpf representative views, there is a relative decrease in inactive phospho-FoxO1 (Ser 256) and FoxO3 (Ser 253). Figure labels: F is femur, T is tibia, M is meniscus. Figure labels: F is femur, T is tibia, M is meniscus. Figure labels: F is femur, T is tibia, M is meniscus.

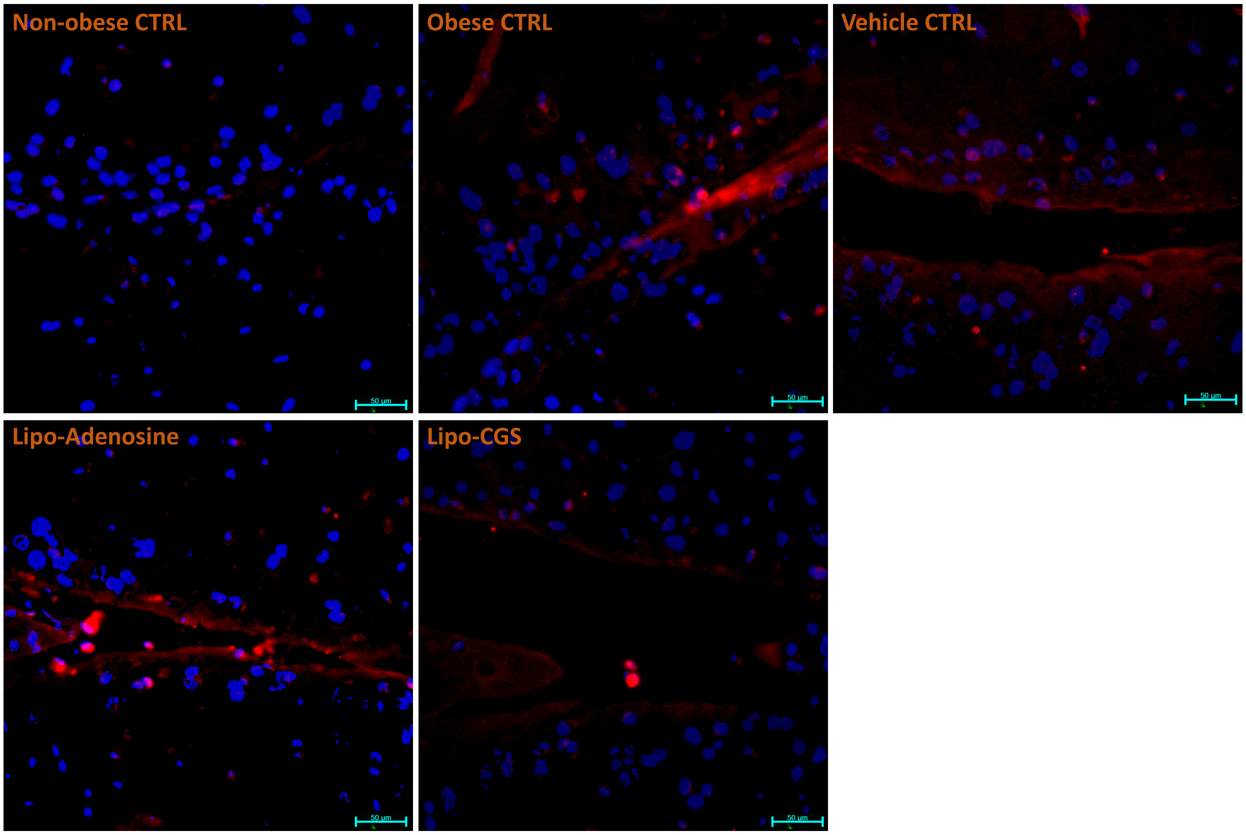

Fig S10. p62 appears to accumulate in untreated obese mice, indicating disruption of autophagy *in vivo*. IHC staining for p62 in the mouse obesity model showing cellular (and some joint line non-specific) fluorescence that appeared to be increased cellularly in the obese and vehicle control. Similar to *in vitro* p62 autophagy studies, one expects an elevation in levels to generally correlate with a block in autophagic flux.
